# RETRACTION: Meta‐Analysis of the Influence of Tracheal Intubation With Cuff and Without Cuff on the Incidence of Total Wound Complications in ICU Intubation Patients

**DOI:** 10.1111/iwj.70532

**Published:** 2025-04-18

## Abstract

**RETRACTION:** JiangB. , WangY. , HeX. , ZhangL. , and FuS. , “Meta‐Analysis of the Influence of Tracheal Intubation With Cuff and Without Cuff on the Incidence of Total Wound Complications in ICU Intubation Patients,” International Wound Journal 21, no. 3 (2024): e14741, 10.1111/iwj.14741.38414304 PMC10899797

The above article, published online on 27 February 2024, in Wiley Online Library (http://onlinelibrary.wiley.com/), has been retracted by agreement between the journal Editor in Chief, Professor Keith Harding; and John Wiley & Sons Ltd. Following an investigation by the publisher, all parties have concluded that this article was accepted solely on the basis of a compromised peer review process. The editors have therefore decided to retract the article. The authors did not respond to our notice regarding the retraction.

**RETRACTION:** B. Jiang , Y. Wang , X. He , L. Zhang , and S. Fu , “Meta‐Analysis of the Influence of Tracheal Intubation With Cuff and Without Cuff on the Incidence of Total Wound Complications in ICU Intubation Patients,” International Wound Journal 21, no. 3 (2024): e14741, 10.1111/iwj.14741.38414304 PMC10899797

The above article, published online on 27 February 2024, in Wiley Online Library (http://onlinelibrary.wiley.com/), has been retracted by agreement between the journal Editor in Chief, Professor Keith Harding; and John Wiley & Sons Ltd. Following an investigation by the publisher, all parties have concluded that this article was accepted solely on the basis of a compromised peer review process. The editors have therefore decided to retract the article. The authors did not respond to our notice regarding the retraction.

